# Painless Caustic

**Published:** 1858-11

**Authors:** 


*Painless Caustic.*—M. Picdagnel, after various trials, has succeeded in producing a caustic that may be employed, causing little or no pain. It is formed of three parts of the Vienna caustic in powder and one part of hydrochlorate of morphia, intimately mixed together, and then made into a thick paste by means of chloroform, alcohol, or water. It is applied to the skin on diachylon. A black eschar is produced in fifteen minutes, increasing in thickness with the duration of the application. The morphia mixed in the same proportions with powdered cantharides, prevents pain during the raising of a blister. M. Picdagnel, who at present has only used this means for the production of issues and blisters, states that the action of the morphia is merely local.—*Gaz. de Hop.*

